# Supplementary material for: Causal Relationship between Aging and Anorexia Nervosa: A White-Matter-Microstructure-Mediated Mendelian Randomization Analysis
Source: Biomedicines. 2024 Aug 16;12(8):1874. doi: 10.3390/biomedicines12081874 (PMC11351342; doi:10.3390/biomedicines12081874)
Supplement: Supplementary file 1 [file biomedicines-12-01874-s001.zip › Supplementary Figure/Figure S4/Singe SNP plot of MR analysis for TL to ubm-b-2084.pdf]

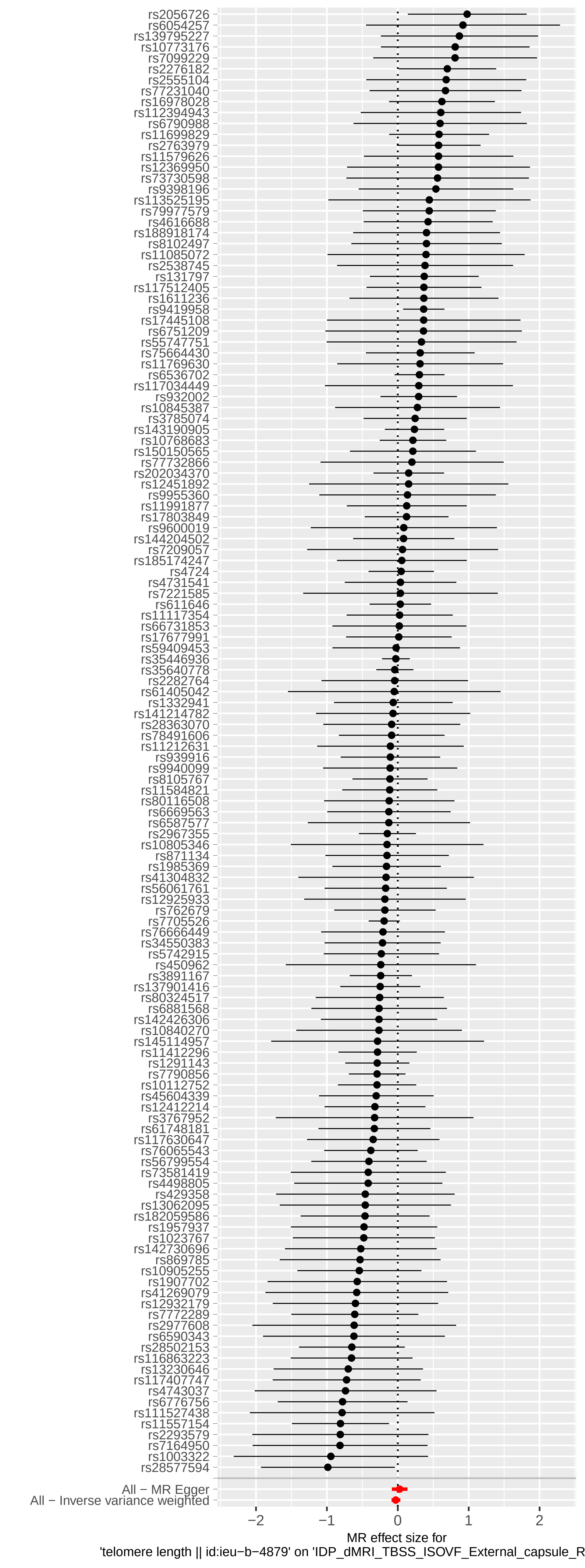
rs2056726
rs6054257
rs139795227
rs10773176
rs7099229
rs2276182
rs2555104
rs77231040
rs16978028
rs112394943
rs6790988
rs11699829
rs2763979
rs11579626
rs12369950
rs73730598
rs9398196
rs113525195
rs79977579
rs4616688
rs188918174
rs8102497
rs11085072
rs2538745
rs131797
rs117512405
rs1611236
rs9419958
rs17445108
rs6751209
rs55747751
rs75664430
rs11769630
rs6536702
rs117034449
rs932002
rs10845387
rs3785074
rs143190905
rs10768683
rs150150565
rs77732866
rs202034370
rs12451892
rs9955360
rs11991877
rs17803849
rs9600019
rs144204502
rs7209057
rs185174247
rs4724
rs4731541
rs7221585
rs611646
rs11117354
rs66731853
rs17677991
rs59409453
rs35446936
rs35640778
rs2282764
rs61405042
rs1332941
rs141214782
rs28363070
rs78491606
rs11212631
rs939916
rs9940099
rs8105767
rs11584821
rs80116508
rs6669563
rs6587577
rs2967355
rs10805346
rs871134
rs1985369
rs41304832
rs56061761
rs12925933
rs762679
rs7705526
rs76666449
rs34550383
rs5742915
rs450962
rs3891167
rs137901416
rs80324517
rs6881568
rs142426306
rs10840270
rs145114957
rs11412296
rs1291143
rs7790856
rs10112752
rs45604339
rs12412214
rs3767952
rs61748181
rs117630647
rs76065543
rs56799554
rs73581419
rs4498805
rs429358
rs13062095
rs182059586
rs1957937
rs1023767
rs142730696
rs869785
rs10905255
rs1907702
rs41269079
rs12932179
rs7772289
rs2977608
rs6590343
rs28502153
rs116863223
rs13230646
rs117407747
rs4743037
rs6776756
rs111527438
rs11557154
rs2293579
rs7164950
rs1003322
rs28577594
All − MR Egger
All − Inverse variance weighted
−2
−1
0
1
2
MR effect size for
'telomere length || id:ieu−b−4879' on 'IDP_dMRI_TBSS_ISOVF_External_capsule_R
